# Studies of Corrosion Inhibition Performance of Inorganic Inhibitors for Aluminum Alloy

**DOI:** 10.3390/ma18030595

**Published:** 2025-01-28

**Authors:** Redouane Farid, Dilip K. Sarkar, Santanu Das

**Affiliations:** 1 University Research Centre on Aluminium (CURAL) and Aluminium Research Centre-REGAL, Department of Applied Science, University of Québec at Chicoutimi (UQAC), 555 Boulevard de l’Université, Chicoutimi, QC G7H 2B1, Canada; farid.redouane@live.fr; 2 Department of Ceramic Engineering, Indian Institute of Technology (BHU), Varanasi 221005, Uttar Pradesh, India; santanudas.cer@iitbhu.ac.in

**Keywords:** aluminum, corrosion inhibition, impedance spectroscopy, polarization resistance, SEM

## Abstract

In this study, the behavior of sodium silicate (Na_2_SiO_3_), manganese sulfate monohydrate (MnSO_4_·H_2_O), and ammonium metavanadate (NH_4_VO_3_) as corrosion inhibitors for AA6061 aluminum alloy (Al) was investigated. The polarization resistance (R_p_) of the Al substrate immersed in 0.1 M NaCl solution was found to be 13 kΩ·cm^2^. In comparison, the R_p_ of the Al substrate immersed in 0.1 M NaCl in the presence of Na_2_SiO_3_, Na_2_SiO_3_/MnSO_4_·H_2_O, and Na_2_SiO_3_/NH_4_VO_3_ inhibitors was found to be 100, 133, and 679 kΩ·cm^2^, respectively. The best inhibition result was obtained when the mixture of the inhibitors was used with R_p_ of 722 kΩ·cm^2^. The maximum percentage of the corroded area calculated from the scanning electron microscopy (SEM) images was found to be 5.7% for Al substrate immersed in 0.1 M NaCl, which decreased to 0.06% when the mixture of the inhibitors was used. The synergetic effects between the three inhibitors were studied, and the results illustrated that the combination of Na_2_SiO_3_, MnSO_4_·H_2_O, and NH_4_VO_3_ provided the best corrosion inhibition properties for Al in aqueous NaCl environments.

## 1. Introduction

Aluminum (Al) and its alloys have several applications in aerospace [1], aircraft [2], automobiles [3], marine industry [4], computer components, food packaging [5], and construction [6], due to their favorable properties such as low density, high ductility, excellent strength-to-weight ratio, and high stiffness [7]. Being a reactive metal, Al reacts with atmospheric oxygen to form a passive and stable protective aluminum oxide (Al_2_O_3_) layer, which provides protection from corrosion in neutral environments [8]. However, Al is susceptible to corrosion in aggressive mediums, such as seawater, which limits its potential applications. Hence, it is essential to explore more corrosion protection strategies to utilize the advantages of Al and its alloys, particularly for applications in marine or highly corrosive environments.

Traditionally, chromate surface treatment technologies [9,10] were used for corrosion protection of Al alloys. However, the chromate ions have raised some health and human safety concerns, resulting in the ban of these technologies in recent times [11]. Thus, researchers need to investigate alternative non-toxic or less toxic corrosion protection techniques. Corrosion inhibition is one such technique, which reduces the corrosion rate of Al when exposed to highly corrosive environments. In this technique, silicate-based materials are investigated for their buffering ability, polymerizing capability, heat and chemical stability [12]. Especially, silicate-based materials are popularly used as inhibitors, which reduce the corrosion rate of metals in a corrosive environment [13,14]. Garrity et al. [13] investigated the corrosion protection of aluminum through the inhibition process by incorporating sodium silicate in the 0.1 M NaCl aqueous solution. They found that the corrosion protection mechanism is due to the formation of aluminosilicate complex that adsorbs on the aluminum surface, further preventing the penetration of corrosive ions.

Furthermore, a frequent contender to chromium is manganese-based alloy compounds. These compounds are studied for their corrosion inhibition properties as manganese exhibits multiple oxidation states similar to chromium. Even though the corrosion inhibition ability of the manganese is not as effective as the latter (Chromium) mainly for low-pH environment, the former (Manganese) inhibits the corrosion of Al at high-pH values [15]. This inhibition is due to the reduction in Mn(VII) in alkaline media, which leads to the formation of MnO_2_, which is considered a less soluble species [16]. In another study, Agnesia et al. [17] reported the corrosion inhibition of Al by potassium permanganate (KMnO_4_) in an alkaline aqueous solution. They found that the corrosion inhibition efficiency increases along with the concentration of KMnO_4_. The formation of the Al-KMnO_4_ complex on the metal surface prevents the penetration of corrosive ions to the substrate and thus increases the corrosion performance of the inhibitor.

Another effective environmental alternative inhibitor to chromium source is vanadium compounds. These compounds have been considered suitable corrosion inhibitors for Al alloys in several studies [18,19,20]. As reported, the inhibition property of vanadium compounds in alkaline aqueous solutions is related to tetrahedrally coordinated V^+5^ anions, such as ${VO}_{4}^{3-}$ and VO(OH)^−2^ [19]. These anions decrease the kinetics of oxygen reduction, which are the dominant cathodic reactions in the corrosion process. In this context, Ralston et al. [18] studied the corrosion inhibition of Al by aqueous vanadium species. They observed that the addition of sodium metavanadate (NaVO_3_) has no effect in an acidic medium but led to a noticeable increase in corrosion protection in an alkaline medium. They concluded that the inhibition of Al by NaVO_3_ in sodium chloride (NaCl) solutions was associated with the formation of tetrahedrally coordinated vanadate. However, the octahedrally coordinated vanadate predominated in the acidic environments does not seem to provide inhibition and may accelerate corrosion under deaerated conditions.

Although studies on the corrosion inhibition of Al by each compound (sodium silicate, manganese, or vanadium) are abundant in the literature, studies on the synergetic effect between the three inhibitors are not yet studied thoroughly. To this end, the present study aims to illustrate the detailed corrosion inhibition mechanisms of sodium silicate (Na_2_SiO_3_), manganese sulfate monohydrate (MnSO_4_·H_2_O), and ammonium metavanadate (NH_4_VO_3_) on AA6061 Al alloy and their subsequent effects on corrosion protection in corrosive environments. An optimized combination of all three inhibitors is also studied to analyze the synergetic effect between the inhibitors, where the corrosion properties of the specimens are investigated using electrochemical tests. The surface morphology and chemical composition of the specimens are also analyzed after the corrosion tests to compare the performance of each inhibitor.

## 2. Experimental

**Figure 1:**
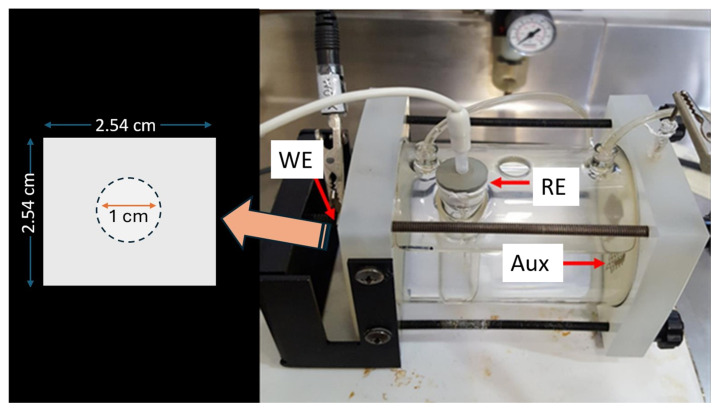
The figure shows a three-electrode electrochemical corrosion cell, including the working electrode (WE) with a 1 cm^2^ exposed area, a reference electrode (RE), and an auxiliary electrode (Aux).

In this study, the AA6061 Al alloy with the chemical composition of Al 97.9 wt.%, Mg 1.08 wt.%, Si 0.63 wt.%, Mn 0.52 wt.%, Cu 0.32 wt.%, Fe 0.17 wt.%, Ti 0.02 wt.%, and V 0.01 wt.% was used as substrates. Each Al substrate, with the size of one by one inch, was ultrasonically degreased in a soap solution and cleaned in ethanol and deionized water for 30 min. The specimens were mounted in the electrochemical cell and exposed to a 1 cm^2^ area of corrosive solutions containing 0.1 M sodium chloride (NaCl, pH 5.9) in the presence of inhibitors (Figure 1).

Three different inhibitors were used, namely sodium silicate (Na_2_SiO_3_) with the concentration of 3 × 10^−2^ M, manganese sulfate monohydrate (MnSO_4_·H_2_O) with the concentration of 3 × 10^−4^ M, and ammonium metavanadate (NH_4_VO_3_) with the concentration of 3 × 10^−4^ M. The inhibitors were added to the NaCl aqueous solution and kept for 24 h to assess their corrosion inhibitive properties on Al using electrochemical studies. For each inhibitor, the tests were repeated in triplicate. Solartron analytical 1252 and SI 1287 potentiostat equipped with a 300 cm^3^ volume flat cells were used to perform the open circuit potential (OCP), electrochemical impedance spectroscopy (EIS), and potentiodynamic polarization (Tafel) tests. A three-electrode set-up, consisting of a platinum mesh (counter electrode), a silver/silver-chloride (Ag/AgCl) (reference electrode), and the Al specimens (working electrodes), was employed for all these electrochemical analyses reported in this study. The frequency range of the EIS studies were fixed between 10^−2^ and 10^5^ Hz, whereas an amplitude of 20 mV was kept with respect to OCP for all the analysis. The potentiodynamic polarization curves were scanned in a range of −300 mV to 1200 mV with respect to OCP. The morphological and elemental characterizations of the specimens were performed by scanning electron microscopy (SEM, JEO JSM-6480 LV, Tokyo, Japan) equipped with energy dispersive X-ray spectroscopy (EDS). Additionally, the image analysis software Clemex (CLEMEX JS-2000, PE4.0, Longueuil, QC, Canada) was used to determine the percentage of the corroded area on Al substrates.

## 3. Results and Discussion

**Figure 2:**
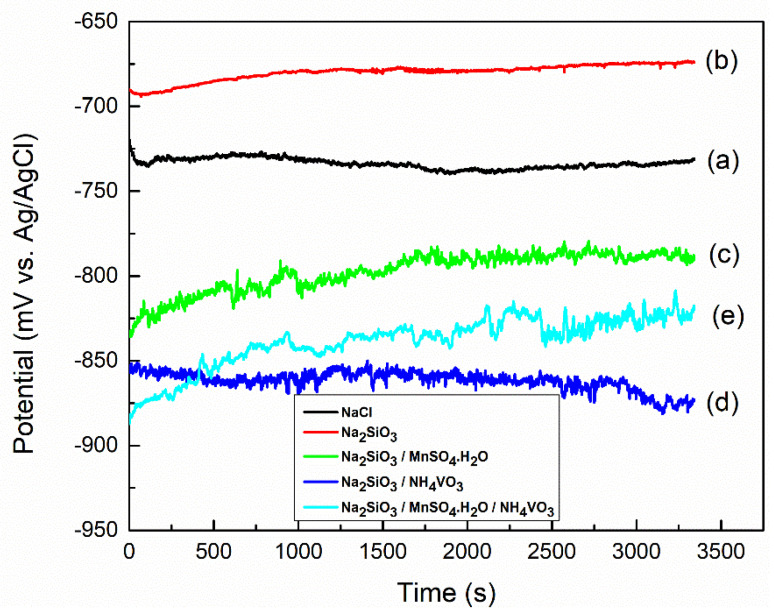
Open circuit potential (OCP) curves of (a) Al substrate immersed in 0.1 M NaCl without inhibitors, and with the presence of (b) Na_2_SiO_3_, (c) Na_2_SiO_3_/MnSO_4_·H_2_O, (d) Na_2_SiO_3_/NH_4_VO_3_, (e) and Na_2_SiO_3_/MnSO_4_·H_2_O/NH_4_VO_3_.

Figure 2 shows the open circuit potential (OCP) curves of the Al substrates immersed in 0.1 M NaCl without inhibitors, and in the presence of Na_2_SiO_3_, Na_2_SiO_3_/MnSO_4_·H_2_O, Na_2_SiO_3_/NH_4_VO_3_, and Na_2_SiO_3_/MnSO_4_·H_2_O/NH_4_VO_3_.

The OCP curve of Al substrate immersed in 0.1 M NaCl solution stabilized at around −730 ± 4 mV, and this value is in good agreement with the values reported in the literature [21,22]. Furthermore, in the presence of Na_2_SiO_3_, the OCP curve in the presence of Na_2_SiO_3_ inhibitor shows a shift toward positive potential values and stabilizes at around −666 ± 11 mV. This shift is related to the anodic inhibition nature of sodium silicate and these results are consistent with the values reported in the literature [13,23,24].

Anodic inhibitors, such as sodium silicate, react with Al^3+^ ions, forming an insoluble film, such as aluminosilicate, which results in a shift in the potential towards more positive values compared to the aluminum substrate in NaCl solution [25].

Contrarily, the OCP curve for the specimen contained in Na_2_SiO_3_/MnSO_4_·H_2_O inhibitors shifted towards a more negative potential (−780 ± 17 mV) compared to −730 ± 4 mV for the Al substrate. Madden et al. reported similar observation on the relative decrease in OCP values [26], where they studied the effect of potassium permanganate (KMnO_2_) on the corrosion properties of Al. They evidenced that the OCP values were reduced to its lower potentials after 24 h of immersion in sodium chloride aqueous solution. The authors attributed the steady drop in the potential due to the reduction in oxidation state of manganese. Furthermore, as seen in Figure 2, the OCP curve for the solution contained the inhibitor of Na_2_SiO_3_/NH_4_VO_3_, stabilized at a further lower value of −854 ± 7 mV. Ralston et al. [27] also reported a similar decreasing tendency of the OCP curve for Al in the presence of NaVO_3_. They evidenced that adding NaVO_3_ in NaCl solution led to a fall in the OCP from −650 mV to −790 mV for the Al substrate. This negative shift, as compared to Al, could be due to the decrease in the dissolution of the intermetallic phases by vanadium, which led to less distributed copper on the surface. In this context, it is pertinent to mention that copper has a positive reduction potential of +340 mV, while aluminum has a negative reduction potential of −1660 mV with respect to standard hydrogen electrode, as given by the Nernst equation. Interestingly, in our experiment, the OCP of the specimen in the presence of Na_2_SiO_3_/MnSO_4_·H_2_O/NH_4_VO_3_ stabilized at the value of −833 ± 5 mV, which is an intermediate value between the OCP curves of the specimens inhibited with Na_2_SiO_3_/MnSO_4_·H_2_O and Na_2_SiO_3_/NH_4_VO_3_.

**Figure 3:**
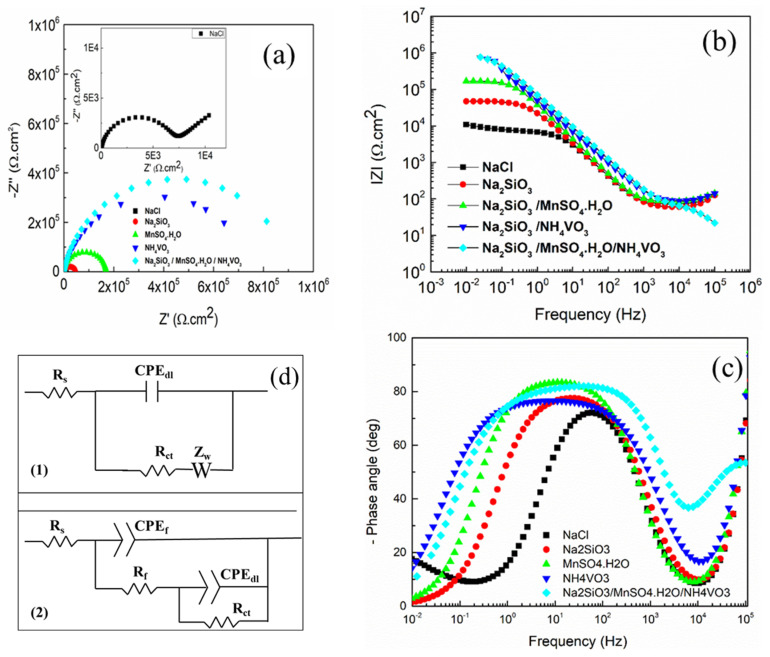
(**a**) Nyquist plots; (**b**) Bode modulus diagrams; (**c**) Bode phase diagrams; and (**d**) equivalent circuit of the Al substrate immersed in 0.1 M NaCl without and with the presence of Na_2_SiO_3_, Na_2_SiO_3_/MnSO_4_·H_2_O, Na_2_SiO_3_/NH_4_VO_3_, and Na_2_SiO_3_/MnSO_4_·H_2_O/NH_4_VO_3_ inhibitors.

Figure 3 shows the EIS measurements of the Al substrates immersed in 0.1 M NaCl solution for 24 h, both in the presence of different inhibitors and their mixtures, namely Na_2_SiO_3_, Na_2_SiO_3_/MnSO_4_·H_2_O, Na_2_SiO_3_/NH_4_VO_3_, and Na_2_SiO_3_/MnSO_4_·H_2_O/NH_4_VO_3_. Figure 3a presents the Nyquist plots of the Al substrate immersed in 0.1 M NaCl and in the presence of Na_2_SiO_3_, Na_2_SiO_3_/MnSO_4_·H_2_O, Na_2_SiO_3_/NH_4_VO_3_, and Na_2_SiO_3_/MnSO_4_·H_2_O/NH_4_VO_3_, which depicts the imaginary component (−Zs) vs the real component (Z’). The Nyquist plot of the Al substrate (magnified in the inset of Figure 3a) is composed of a small semi-circle at high frequencies followed by a linear part at low frequencies. The semi-circle indicates the charge transfer resistance (R_ct_) of 6.7 kΩ·cm^2^, while the linear part at low frequencies in the Nyquist plot corresponds to the Warburg impedance, which is primarily due to the diffusion of the electrolyte into the Al substrate [28]. On the contrary, with inhibitors of Na_2_SiO_3_, Na_2_SiO_3_/MnSO_4_·H_2_O, Na_2_SiO_3_/NH_4_VO_3_, and Na_2_SiO_3_/MnSO_4_·H_2_O/NH_4_VO_3_ in 0.1 M NaCl solution, the Nyquist plots show only one semi-circle.

A relatively higher R_ct_ value of 47 kΩ·cm^2^ was observed for aluminum in the presence of Na_2_SiO_3_ as an inhibitor. Such an increase in the Rct value could be due to forming a protective layer of aluminosilicate, which acts as a barrier to oxygen diffusion into the metallic surface [29]. Furthermore, in the presence of Na_2_SiO_3_/MnSO_4_·H_2_O, the R_ct_ has further increased to 169 kΩ·cm^2^.

In a similar terrain, Mohammadi et al. [30] studied the inhibition performance of potassium permanganate (KMnO_4_) on Al in 3.5 wt.% (0.6 M) NaCl solution. They found that the R_ct_ value raised from 5.5 to 27.2 kΩ·cm^2^ as the concentration of KMnO_4_ increased from 0.01 M to 0.1 M. The lower reported values of R_ct_, in their study, compared to the values reported in our study could be due to the difference in the concentration of the NaCl solution.

Furthermore, the presence of Na_2_SiO_3_/NH_4_VO_3_ as an inhibitor in 0.1 M NaCl solution resulted in a much higher R_ct_ of 753 kΩ·cm^2^, which indicates that the vanadium-based inhibitor has good protection against corrosion in the presence of sodium silicate. In this context, it is worth mentioning the study of Kharitonov et al. [19], in which they reported the two-step inhibition process of vanadium-based inhibitors in an alkaline environment. In the first step, the V^+5^ vanadates were reduced to V^+4^ or V^3+^ species on the surface of the cathodic intermetallic phases and formed mixed-valence V^+5^/V^+4^ polymerized compounds. Then, in the second step, these compounds were precipitated on the Al surface to form a thin protective layer on the substrate [19]. The highest R_ct_ of 926 kΩ·cm^2^ were achieved for aluminum in the presence of the mixture of inhibitors of Na_2_SiO_3_/MnSO_4_·H_2_O/NH_4_VO_3_. These results could be due to a synergetic effect between the inhibitors, leading to a complex protective layer on the Al surface.

**Figure 4:**
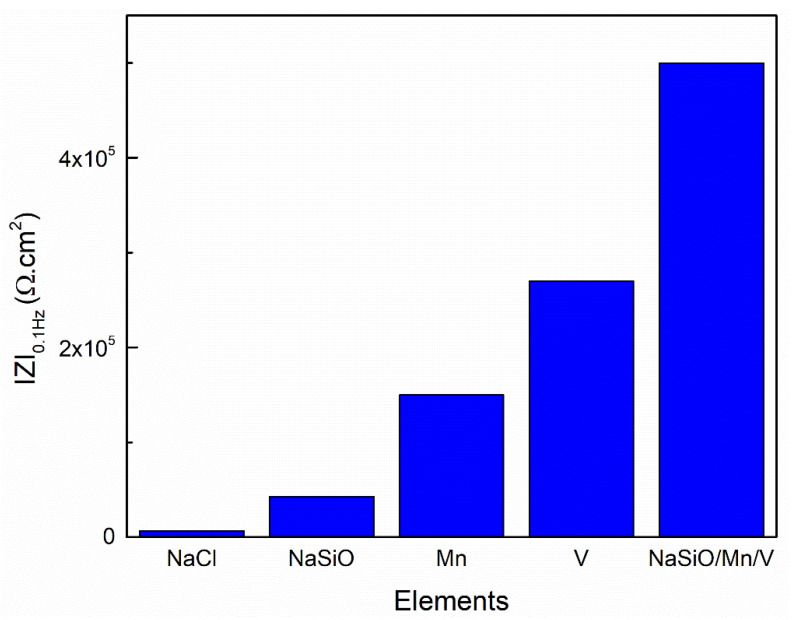
The impedance |Z| at the frequency of 0.1 Hz of the Al substrate immersed in 0.1 M NaCl solution and with the presence of Na_2_SiO_3_, Na_2_SiO_3_/MnSO_4_·H_2_O, Na_2_SiO_3_/NH_4_VO_3_, and Na_2_SiO_3_/MnSO_4_·H_2_O/NH_4_VO_3_.

Figure 3b shows the Bode modulus diagrams of the Al substrate in the aqueous solution of 0.1 M NaCl without and with the presence of Na_2_SiO_3_, Na_2_SiO_3_/MnSO_4_·H_2_O, Na_2_SiO_3_/NH_4_VO_3_, and Na_2_SiO_3_/MnSO_4_·H_2_O/NH_4_VO_3_. The impedance |Z| has been presented with respect to frequency. It is well known that the inhibition effects are usually identified by the impedance values at low frequencies (e.g., 0.1 Hz), as inhibition is often related to the suppression of electron transfer caused by forming a passive layer on the Al substrate [31,32]. Thus, to evaluate the effectiveness of the inhibitors’ corrosion protection, the impedance values at the low-frequency value of 0.1 Hz are plotted and presented in Figure 4.

The impedance |Z| for Al substrate in 0.1 M NaCl solution was found to be 0.7 kΩ·cm^2^, which increased systematically to 42, 150, 270, and 500 kΩ·cm^2^ for the Al substrates with the presence of Na_2_SiO_3_, Na_2_SiO_3_/MnSO_4_·H_2_O, Na_2_SiO_3_/NH_4_VO_3_, and Na_2_SiO_3_/MnSO_4_·H_2_O/NH_4_VO_3_ inhibitors, respectively.

The imaginary part of the impedance is inversely proportional to the capacitance of the passivated layers and is given by

(1) $$Z^{\prime \prime }=\frac{1}{j\omega C},$$

where Z″ is the imaginary part of the impedance, j=√−1 is the imaginary number, ω is the angular frequency (ω = 2πƒ, ƒ is the frequency), and C is the capacitance of the passivated layer.

Hence, at the low-frequency region (0.01 to 1 Hz), the total modulus is dominated by 1/ωC. On the other hand, the capacitance of the passivated layer is a function of its dielectric constant, and its thickness is given by

(2) $$C=\varepsilon_{0}\varepsilon \frac{A}{d}\;,$$

where *A* is the effective electrode surface area, which is constant in our experiment. ε_0_ is the vacuum permittivity (8.85 × 10^−14^ F/cm) and ε and *d* are the dielectric constant and the thickness of the passivated layer, respectively. As a result, the impedance modules at low frequency are proportional to the thickness (*d*) and inversely proportional to the dielectric constant ε, as shown in Equation (3).

(3) $$Z^{\prime \prime }=\left(\frac{1}{j\omega A\varepsilon_{0}}\right)\left(\frac{d}{\varepsilon }\right)=\alpha \left(\frac{d}{\varepsilon }\right)$$

**Figure 5:**
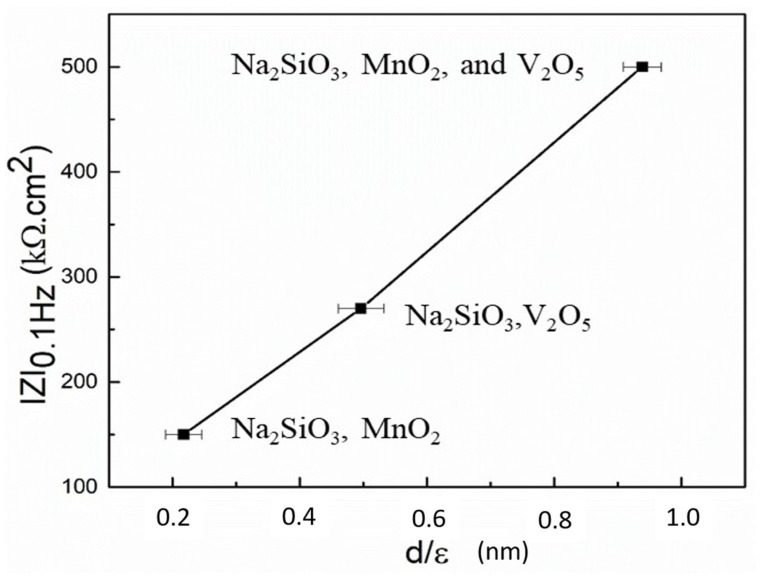
The variation in the impedance |Z| at the frequency of 0.1 Hz as a function of d/ε of the probable adsorbed oxides on the Al substrate during the inhibition process.

As the thickness of the passivated layer on Al substrate is very small in the presence of different inhibitors, the ATR-FTIR absorption intensity is used as the representative of thickness. The passivated layer on the Al substrate is composed of oxides of Na_2_SiO_3_, MnO_2_, and V_2_O_5_ inhibitors, and their dielectric constants are 9, 72, and 36, respectively [33,34,35]. The graph of |Z| vs. d/ε has been plotted and presented in Figure 5. The linear relationship between |Z| and d/ε indicates the synergetic effect among the inhibitors. On the contrary, the Al substrate in the presence of Na_2_SiO_3_ as an inhibitor does not satisfy the linear correlation, which could be due to the lower thickness of the protective layer on the Al substrate.

Furthermore, Figure 3c describes the Bode phase diagram of the Al substrate in the aqueous solution of 0.1 M NaCl with and without the presence of Na_2_SiO_3_, Na_2_SiO_3_/MnSO_4_·H_2_O, Na_2_SiO_3_/NH_4_VO_3_, and Na_2_SiO_3_/MnSO_4_·H_2_O/NH_4_VO_3_ inhibitors. The full width at half maximum (FWHM) of the phase bands was calculated using analysis software, and the results are summarized in Table 1. The FWHM value of 164 Hz is found for the Al substrate immersed in 0.1 M NaCl. Furthermore, FWHM values increased systemically to 1905, 3019, 26,001, and 27,227 Hz for the specimen in the presence of Na_2_SiO_3_, Na_2_SiO_3_/MnSO_4_·H_2_O, Na_2_SiO_3_/NH_4_VO_3_, and Na_2_SiO_3_/MnSO_4_·H_2_O/NH_4_VO_3_ inhibitors, respectively. The increase in the FWHM values with the addition of the inhibitors can be related to the adsorption process of inhibitors on the Al substrate, which leads to a change in the local dielectric constant [19]. On the other hand, the maximum phase angle shifts towards the lower frequencies in the presence of the inhibitors (Na_2_SiO_3_, Na_2_SiO_3_/MnSO_4_·H_2_O, Na_2_SiO_3_/NH_4_VO_3_, and Na_2_SiO_3_/MnSO_4_·H_2_O/NH_4_VO_3_). This shift can be associated with the change in the local dielectric constant of the thin film, resulting from the probable deposition of Na_2_SiO_3_, VO_2_, and MnO_2_ with their respective dielectric constants of 9, 36, and 72 [33,34,35].

Figure 3(d1,d2) show the electrical equivalent circuits (EEC) for the experimental impedance spectroscopy data of Al substrate immersed in 0.1 M NaCl with and without the presence of Na_2_SiO_3_, Na_2_SiO_3_/MnSO_4_·H_2_O, Na_2_SiO_3_/NH_4_VO_3_, and Na_2_SiO_3_/MnSO_4_·H_2_O/NH_4_VO_3_ inhibitors. Figure 3(d1) shows the EEC of the Al substrate immersed in 0.1 M NaCl solution, which is represented by an ohmic resistance (R_S_) connected in series with Resistance capacitance (RC) circuit composed of capacitance C_1_ and a charge transfer resistance (R_ct_) in parallel. The circuit also contains a Warburg factor element (Z_w_) that indicates the diffusion process of the electrolyte/coating interface.

Furthermore, the constant phase element (CPE) is used instead of ideal electrical capacitance in the circuits for the specimens with inhibitors due to the existence of heterogeneity in its microstructure and chemical composition [36]. Figure 3(d2) represents the EEC of the specimens with inhibitors, where R_S_ is connected with two RC circuits in series. CPE_f_ represents the CPE related to the dielectric properties of the specimens, and R_f_ represents the resistance provided by the inhibition film on the Al surface. On the other hand, CPE_dl_ denotes the CPE associated with the double layer at the interface near the Al surface, while R_ct_ is the charge transfer resistance. Table 2 summarizes the impedance parameters of EEC for the Al substrate immersed in 0.1 M NaCl and Table 3 gives the values of the fitted parameter of the equivalent circuit of EIS measurements of Al substrate immersed in 0.1 M for 24 h in the presence of Na_2_SiO_3_, Na_2_SiO_3_/MnSO_4_·H_2_O, Na_2_SiO_3_/NH_4_VO_3_, and Na_2_SiO_3_/MnSO_4_·H_2_O/NH_4_VO_3_ inhibitors.

Notably, from Table 3, the CPE_dl_ of the Al substrate immersed in 0.1 M NaCl in the presence of Na_2_SiO_3_, Na_2_SiO_3_/MnSO_4_·H_2_O, Na_2_SiO_3_/NH_4_VO_3_, and Na_2_SiO_3_/MnSO_4_·H_2_O/NH_4_VO_3_ are found to be 6.99 × 10^−6^, 4.38 × 10^−6^, 4.02 × 10^−6^, and 2.29 × 10^−6^ Ω^−1^·S^n^·cm^−2^, respectively, which indicate a systematic decrease in CPE_dl_ in the case of using the aforementioned mixture of inhibitors sequentially. This systematic decrease in CPE_dl_ indicates that the hydrogen evolution reactions on the Al substrate are decelerated, suggesting a better inhibition performance when the mixture of the inhibitors is used in the solution [37].

**Figure 6:**
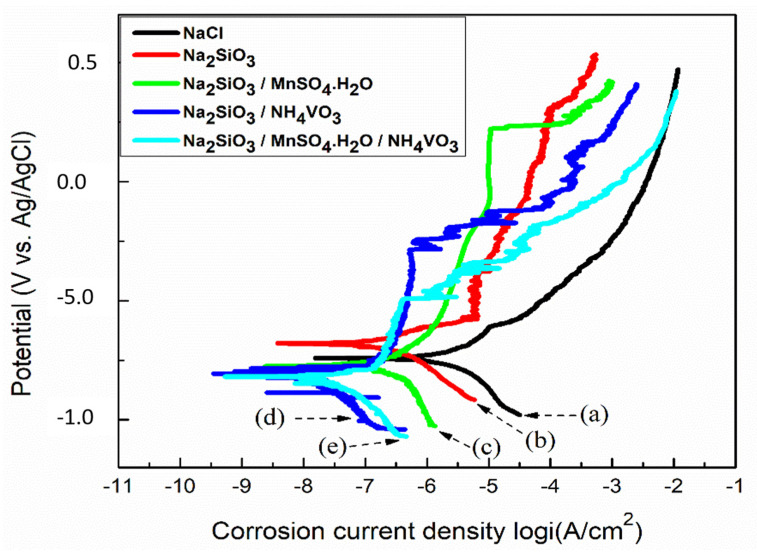
Potentiodynamic polarization curves of (a) Al substrate immersed in 0.1 M NaCl for 24 h without inhibitors, and with the presence of (b) Na_2_SiO_3_, (c) Na_2_SiO_3_/MnSO_4_·H_2_O, (d) Na_2_SiO_3_/NH_4_VO_3_, (e) and Na_2_SiO_3_/MnSO_4_·H_2_O/NH_4_VO_3_.

Potentiodynamic polarization curves of the Al substrate immersed in 0.1 M NaCl and with the presence of Na_2_SiO_3_, Na_2_SiO_3_/MnSO_4_·H_2_O, Na_2_SiO_3_/NH_4_VO_3_, and Na_2_SiO_3_ or/MnSO_4_·H_2_O/NH_4_VO_3_ as inhibitors are shown in Figure 6.

The polarization resistance (R_p_)is calculated from the Stern-Geary equation as given below:

(4) $$R_{p}=\frac{\beta_{a}\beta_{c}}{2.3I_{corr}(\beta_{a}+\beta_{c})}$$

where β_a_ and β_c_ are the anodic and cathodic slopes of the Tafel curves, respectively. I_corr_ is the corrosion current density. The calculated values of I_corr_, R_p_, and the corrosion potential (E_corr_) for all specimens are presented in Table 4.

Our estimation reveals that the corrosion current density (I_corr_) is highest (1.3 ± 0.6 µA/cm^2^) in the case of the primary specimen when Al substrate immersed in 0.1 M NaCl solution, and polarization resistance (R_p_) (22 ± 9 kΩ·cm^2^) becomes its lowest. The I_corr_ value decreases and the R_p_ value increases systematically as we add mixtures of inhibitors sequentially. For the specimen containing Na_2_SiO_3_ as an inhibitor, the I_corr_ value decreases to 0.4 ± 0.1 µA/cm^2,^ and the R_p_ value increases to 73 ± 26 kΩ·cm^2^. It can be attributed to the formation of a protective layer of aluminosilicate on the Al substrate. This result complements the EIS studies. Garrity et al. [13] have investigated the corrosion protection of Al by adding different concentrations of Na_2_SiO_3_ as an inhibitor into 0.1 M NaCl aqueous solution. The potentiodynamic polarization curves show that sodium silicate provides strong anodic inhibition.

The specimen contains Na_2_SiO_3_/MnSO_4_·H_2_O as an inhibitor displays a further decrease in I_corr_ value to 0.18 ± 0.02 µA/cm^2^ and an increase in R_p_ to 126 ± 7 kΩ·cm^2^. It has been reported previously [15,16] that Mn-based inhibitors can reduce the corrosion of Al alloys in alkaline solution (pH > 11), which could result from the reduction in Mn(VII) in alkaline environments and the formation of a protective layer of MnO_2_, a less soluble species, on the Al substrate.

The I_corr_ value has further decreased to 0.005 ± 0.002 µA/cm^2^ with an increase in R_p_ to 545 ± 133 kΩ·cm^2^ for the specimen containing the inhibitor Na_2_SiO_3_/NH_4_VO_3_. In a similar study, Ralston et al. [18] investigated the corrosion inhibition of Al by sodium metavanadate (NaVO_3_) at different pH environments. They observed that adding NaVO_3_ does not affect the pH of 3 and 5. However, it leads to a noticeable decrease in corrosion potential and corrosion current density at pH 8 and 10. Their pH values are comparable with the measured pH value of 11 of our specimens containing Na_2_SiO_3_/NH_4_VO_3_ as an inhibitor, demonstrating the better corrosion inhibition properties of Na_2_SiO_3_/NH_4_VO_3_.

Finally, when we add Na_2_SiO_3_/MnSO_4_·H_2_O/NH_4_VO_3_ mixture in our specimen, the I_corr_ value reaches its lowest (0.004 ± 0.002 µA/cm^2^), and R_p_ becomes highest (660 ± 62 kΩ·cm^2^). Thus, the specimen contains a mixture of inhibitors of Na_2_SiO_3_/MnSO_4_·H_2_O/NH_4_VO_3_, providing the best corrosion protection for the Al substrate. The improved corrosion protection properties indicate that the inhibitors display a synergetic effect.

**Figure 7:**
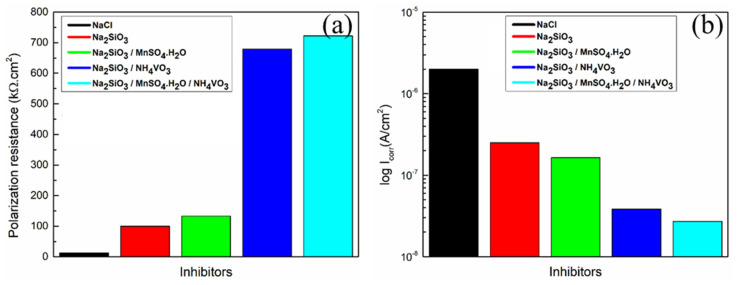
The variation in the (**a**) polarization resistance R_p_ and the (**b**) corrosion current density i_corr_ of the Al substrate immersed in 0.1 M NaCl without inhibitors, and with the presence of Na_2_SiO_3_, Na_2_SiO_3_/MnSO_4_·H_2_O, Na_2_SiO_3_/NH_4_VO_3_, and Na_2_SiO_3_/MnSO_4_·H_2_O/NH_4_VO_3_.

Figure 7a,b show a bar chart comparison of the polarization resistance R_p_ and the corrosion current density I_corr_ of the Al substrate immersed in 0.1 M NaCl solution and with the presence ofNa_2_SiO_3_, Na_2_SiO_3_/MnSO_4_·H_2_O, Na_2_SiO_3_/NH_4_VO_3_, and Na_2_SiO_3_/MnSO_4_·H_2_O/NH_4_VO_3_ as inhibitors. A significant rise in R_p_ value is observed (Figure 7a) for the specimen that contained Na_2_SiO_3_/NH_4_VO_3_ as an inhibitor. A systematic decrease of I_corr_ values is observed (Figure 7b) for specimens in the presence of inhibitors. The specimen contains mixed Na_2_SiO_3_/MnSO_4_·H_2_O/NH_4_VO_3_ as an inhibitor, providing the best corrosion protection for the Al substrate.

Furthermore, corrosion inhibition efficiency (η) (column 5, Table 4) indicates the proportionate deviation of all the specimens was evaluated using the standard equation

(5) $$\eta (\%)=\frac{I_{corr(0)}-I_{corr(inh)}}{I_{corr(0)}}\times 100$$

where I_corr(0)_ is the corrosion current density of Al substrate immersed in 0.1 M NaCl and I_corr(inh)_ is the corrosion current density of the Al substrate in the presence of inhibitors. A systematic increase in η values of 69.2, 86.1, 99.6, and 99.7% are found for the specimens in the presence of Na_2_SiO_3_, Na_2_SiO_3_/MnSO_4_·H_2_O, Na_2_SiO_3_/NH_4_VO_3_, and Na_2_SiO_3_/MnSO_4_·H_2_O/NH_4_VO_3_, respectively. The highest corrosion inhibition efficiency observed in mixed Na_2_SiO_3_/MnSO_4_·H_2_O/NH_4_VO_3_ specimen could be due to the formation of a complex inhibitor structure, which could adsorb on the metallic substrate and prevent the penetration of corrosive ions to the substrate.

To understand synergetic effects for the mixed Na_2_SiO_3_/MnSO_4_·H_2_O/NH_4_VO_3_ specimen (which is likely to be taken place between Na_2_SiO_3_, MnSO_4_·H_2_O, and NH_4_VO_3_ inhibitors), the synergism parameters are calculated using a modified version of the formula proposed by Aramaki et al. in 1969 [38], as presented in Equation (6).

(6) $${s=100-(\eta }_{A+}\eta_{B}-\eta_{AB})$$

where η_A_ and η_B_ are the corrosion inhibition efficiencies calculated for inhibitors A and B, respectively, while η_AB_ is the corrosion inhibition efficiency for the mixture of A and B.

When the inhibitor A or B have no effect on each other and adsorbs independently at the metal/solution interface, then the value of s will be 1. On the other hand, s > 1 represents a manifestation of synergetic effects, while antagonistic effects are characterized when s < 1.

In this study, the synergism parameter, *s*, for the mixed Na_2_SiO_3_/MnSO_4_·H_2_O/NH_4_VO_3_, was found to be 7.8, considering A as Na_2_SiO_3_/MnSO_4_·H_2_O and B as Na_2_SiO_3_/NH_4_VO_3_. This value confirms that the enhanced inhibition efficiency for the mixed inhibitor specimen is achieved due to the synergetic effect.

**Figure 8:**
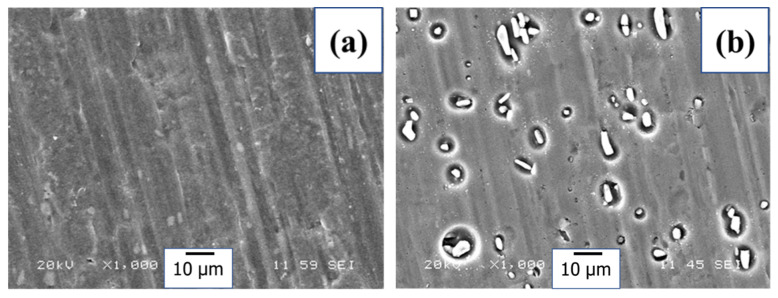

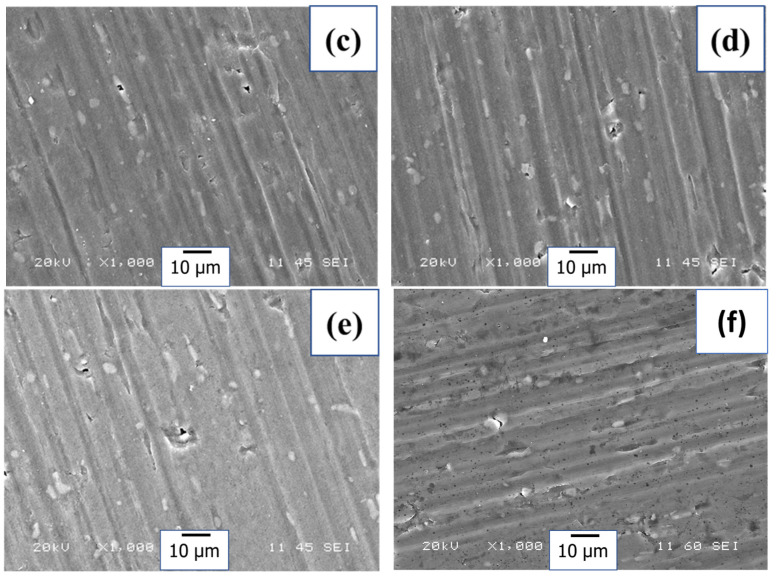
SEM images of (**a**) as-received Al substrate. (**b**) Al substrate immersed in 0.1 M NaCl for 24 h, and with the presence of the inhibitors of (**c**) Na_2_SiO_3_, (**d**) Na_2_SiO_3_/MnSO_4_·H_2_O, (**e**) Na_2_SiO_3_/NH_4_VO_3_, (**f**) and Na_2_SiO_3_/MnSO_4_·H_2_O/NH_4_VO_3_.

Figure 8a represents the SEM image of the as-received substrate; the lines appearing on the substrate surface are due to the rolling process. The SEM image of the Al substrate immersed in 0.1 M NaCl aqueous solution for 24 h is shown in Figure 8b. The image exhibits corrosion-related features leading to exposure of approximately 10 µm × 2 µm size of the intermetallic particles. The potential difference between the elements, such as Mn, of these intermetallic particles and the Al matrix causes the formation of galvanic corrosion cells and leads to localized corrosion of the Al matrix [1].

It is observed from the images that the shape of the corroded area on the Al surface is either circular or elliptical. This shape seems to be determined by the shape of the intermetallic phases. Additionally, the image can be used to determine the percentage of the corroded area of the Al surface using image analysis software, which gave a value of 5.7%. Interestingly, in the image shown in Figure 8c, no such corrosion features are visible for the specimen with Na_2_SiO_3_ as an inhibitor. The percentage of the corroded area is 0.29%. The possible reason behind this percentage reduction is the formation of a sodium silicate protective layer on the Al substrate, which prevents the penetration of corrosive ions to the substrate [13]. A similar image is shown in Figure 8d for the specimen containing Na_2_SiO_3_/MnSO_4_·H_2_O as an inhibitor. The calculated percentage of corrosion is 0.08%. Here, the corrosion has decreased due to the inhibitive properties of Mn in alkaline environments. Mikhailovskii et al. [15], in a similar kind of study, reported that manganese ions (${MnO}_{4}^{-})$ inhibit the corrosion of Al alloys at an alkaline medium due to the formation of the non-soluble species MnO_2_.

**Figure 9:**
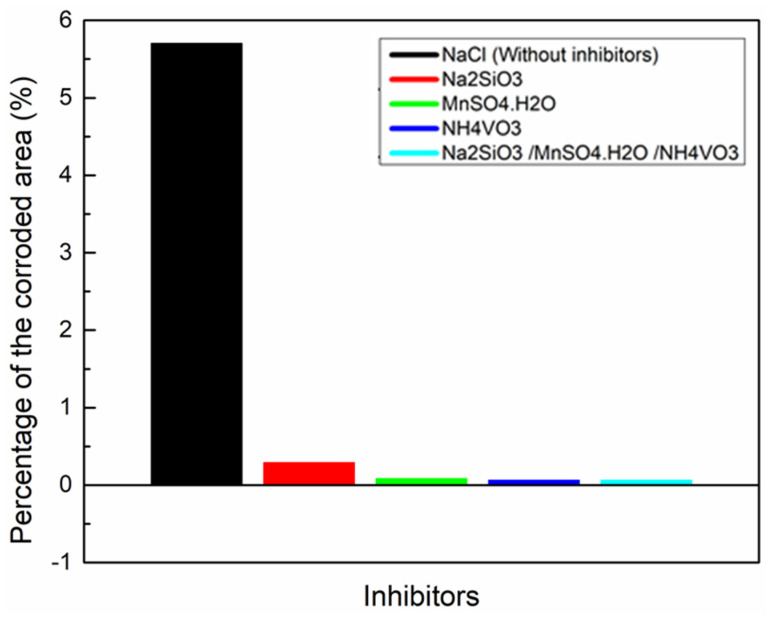
The percentage of the corroded area, determined from SEM images, of the Al substrate immersed in 0.1 M NaCl for 24 h, and with the presence of the inhibitors of Na_2_SiO_3_, Na_2_SiO_3_/MnSO_4_·H_2_O, Na_2_SiO_3_/NH_4_VO_3_, and Na_2_SiO_3_/MnSO_4_·H_2_O/NH_4_VO_3_.

Furthermore, the corroded area decreased to 0.06% with Na_2_SiO_3_/NH_4_VO_3_ as an inhibitor (Figure 8e). Ralston et al. [19] studied the corrosion inhibition of Al by aqueous vanadium species. They found that in alkaline solutions, vanadates play a role in preventing the dissolution of magnesium from the intermetallic particles. As a result, these particles may not become strong cathodes, reducing the localized corrosion’s effect. Figure 8f shows the SEM image of the specimen containing Na_2_SiO_3_/MnSO_4_·H_2_O/NH_4_VO_3_ inhibitor. The calculated percentage of the corroded area was found to be 0.06%. This result indicates an excellent corrosion protection performance for the mixed inhibitors solution due to the formation of a complex inhibitors structure that adsorbs on the Al surface as a protective layer against corrosion. The calculated percentage values of the corroded area for all specimens are presented in Figure 9.

**Figure 10:**
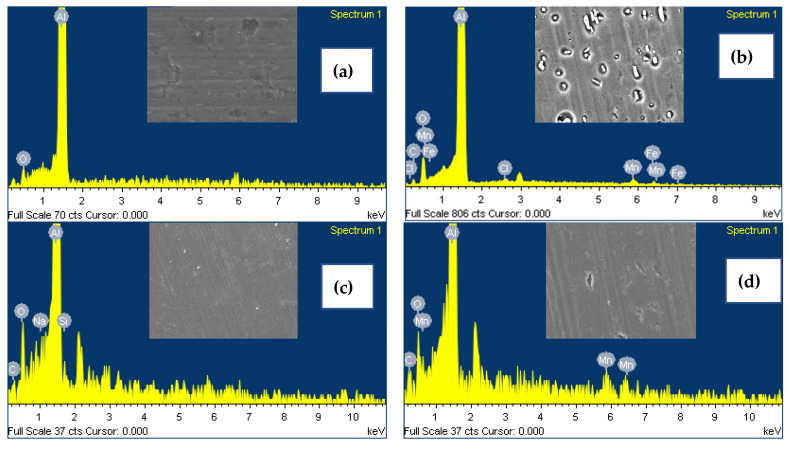

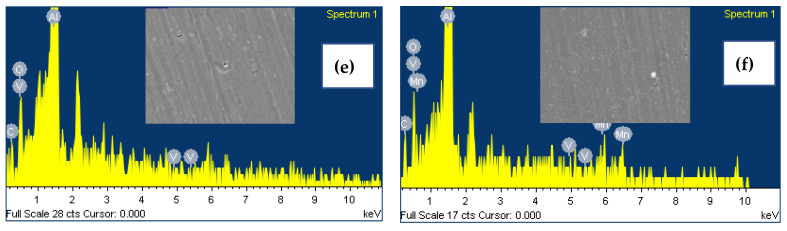
EDS spectra of (**a**) as-received Al substrate, (**b**) Al substrate immersed in 0.1 M NaCl for 24 h, and with the presence of the (**c**) Na_2_SiO_3_, (**d**) Na_2_SiO_3_/MnSO_4_·H_2_O, (**e**) Na_2_SiO_3_/NH_4_VO_3_, and (**f**) Na_2_SiO_3_/MnSO_4_·H_2_O/NH_4_VO_3_ inhibitors. The insets show the corresponding SEM images.

In Figure 10a, the EDS spectra of the as-received Al substrate is characterized by the presence of the elements of O and Al with their respective K_α_ peaks at 0.52 and 1.48 keV. Figure 10b represents the spectrum of Al substrate immersed for 24 h in 0.1 M NaCl solution. It shows the presence of C, O, Al, and Cl with their respective K_α_ peaks at 0.28, 0.52, 1.48, and 2.62 keV. The Lα peaks of Mn and Fe are at 0.63 and 0.70 keV, respectively. The M_α_ peaks of Mn and Fe are located at 5.89 and 6.39 KeV, respectively. The presence of Mn and Fe elements in the surface chemical composition indicates the effect of the localized corrosion process caused by the potential difference between the intermetallic phases and the Al matrix. Figure 10c is the spectrum of the Al substrate immersed for 24 h in 0.1 M NaCl solution in the presence of the Na_2_SiO_3_ inhibitor. The spectrum is composed of C, O, Na, and Si elements with their respective K_α_ peaks at 0.27, 0.52, 1.04, and 1.73 keV, respectively. The presence of Na and Si peaks could be related to the formation of an inhibitive layer of sodium silicate on the Al substrate. Figure 10d is the spectrum of the specimen in the presence of Na_2_SiO_3_/MnSO_4_·H_2_O as an inhibitor. The spectrum is composed of C, O, and Mn elements with their respective K_α_ peaks at 0.27, 0.52, and 5.89 keV, respectively. The Mn-related peak at 0.63 keV, attributed to Lα, could be associated with the formation of sodium silicate—a protective silicate-rich layer capable of incorporating manganese ions, thereby enhancing corrosion protection. Figure 10e indicates the spectrum of the specimen in the presence of the Na_2_SiO_3_/NH_4_VO_3_ inhibitor. This spectrum is composed of C, O and V elements with their respective K_α_ peaks at 0.27, 0.52, and 4.94 keV, respectively. The vanadium L_α_ peak at 0.51 keV could be due to the deposition of vanadium on the Al surface. The presence of these elements indicates the formation of a vanadium-incorporated sodium silicate protective layer. The spectrum in Figure 10f corresponds to the specimen in the presence of Na_2_SiO_3_/MnSO_4_·H_2_O/NH_4_VO_3_. The spectrum is composed of C, O, V, and Mn elements with their respective K_α_ X-ray peaks at 0.2, 0.5, 4.9, and 5.8 keV. The appearance of V and Mn peaks in the EDS spectrum could be associated with the formation of a complex inhibitor layer, which enhances corrosion resistance by effectively blocking the penetration of corrosive ions into aluminum substrates. It is noteworthy to mention that in some instances, the gold conductive coating was applied on specimens for better imaging and analysis, which resulted in the appearance of the peak at around 2.12 keV.

## 4. Conclusions

A comparative study on the corrosion inhibition performance of Na_2_SiO_3_, Na_2_SiO_3_/MnSO_4_·H_2_O, and Na_2_SiO_3_/NH_4_VO_3_ inhibitors for AA6061 aluminum alloy was undertaken. It was found that the mixture of these three inhibitors provided the best performance against corrosion in aqueous NaCl solution. The inhibition efficiency (η) of the inhibitors, calculated from the corrosion current density, was found to be 86.1%, 99.6%, and 99.7% for Na_2_SiO_3_, Na_2_SiO_3_/MnSO_4_·H_2_O, and Na_2_SiO_3_/NH_4_VO_3_, respectively. However, due to the synergistic effect, a maximum efficiency (η) value of 99.9% was achieved with the mixed inhibitors Na_2_SiO_3_/MnSO_4_·H_2_O/NH_4_VO_3_.

To enhance the understanding of these inhibitors’ performance, future studies could investigate their behavior under varying concentrations of the inhibitors, solution pH levels, and experimental temperatures, as these parameters are critical factors influencing inhibitor efficiency. Furthermore, an additional surface-sensitive characterization tool, such as X-ray photoelectron spectroscopy (XPS), could provide a detailed understanding of the composition formed on aluminum surfaces when exposed to these inhibitors. The inhibitors evaluated in this study offer an effective and environmentally friendly alternative to traditional chromate-based inhibitors, potentially advancing corrosion protection strategies in industrial applications.

## Figures and Tables

**Table 1:** Full width at half maximum (FWHM) values calculated from the fitting of Bode phase diagrams in Figure 3c.

Solution	FWHM (Hz)
NaCl	164
Na_2_SiO_3_	1905
Na_2_SiO_3_/MnSO_4_·H_2_O	3019
Na_2_SiO_3_/NH_4_VO_3_	26,001
Na_2_SiO_3_/MnSO_4_·H_2_O/NH_4_VO_3_	27,227

**Table 2:** Fitted parameters of the equivalent circuit of EIS measurements of Al substrate immersed in 0.1 M NaCl for 24 h.

Solution	Rs (Ω·cm^2^)	R_ct_ (kΩ·cm^2^)	C_1_ (µF·cm^−2^)	n_f_	Z_w_ (Ω·cm^2^·S^1/2^)
NaCl	79	6	5.95	0.47	7813

**Table 3:** Fitted parameters of the equivalent circuit of EIS measurements of Al substrate immersed in 0.1 M NaCl for 24 h in the presence of Na_2_SiO_3_, Na_2_SiO_3_/MnSO_4_·H_2_O, Na_2_SiO_3_/NH_4_VO_3_, and Na_2_SiO_3_/MnSO_4_·H_2_O/NH_4_VO_3_ inhibitors.

Solution	Rs (Ω·cm^2^)	CPE_f_		R_f_ (Ω·cm^2^)	CPE_dl_		R_ct_ (kΩ·cm^2^)
		Y_1_ (Ω^−1^·S^n^·cm^−2^)	n_f_		Y_dl_ (Ω^−1^·S^−n^·cm^−2^)	n_dl_	
Na_2_SiO_3_	86	3.26 × 10^−11^	1.40	143	6.99 × 10^−6^	0.90	47
Na_2_SiO_3_/MnSO_4_·H_2_O	99	1.4 × 10^−10^	1.29	183	4.38 × 10^−6^	0.95	169
Na_2_SiO_3_/NH_4_VO_3_	92	1.18 × 10^−10^	1.31	171	4.02 × 10^−6^	0.86	753
Na_2_SiO_3_/MnSO_4_·H_2_O/NH_4_VO_3_	90	2.67 × 10^−6^	0.69	170	2.29 × 10^−6^	0.88	926

**Table 4:** Open circuit potential (OCP), corrosion potential (E_corr_), corrosion current density (I_corr_), polarization resistance (R_p_), and the corrosion inhibition efficiency η (%) of Al substrate immersed in 0.1 M NaCl without inhibitors, and with the presence of Na_2_SiO_3_, Na_2_SiO_3_/MnSO_4_·H_2_O, Na_2_SiO_3_/NH_4_VO_3_, and Na_2_SiO_3_/MnSO_4_·H_2_O/NH_4_VO_3_.

	1	2	3	4	5
	OCP (mV) vs. Ag/AgCl	Corrosion Potential E_corr_ (mV) vs. Ag/AgCl	Corrosion Current Density I_corr_ (µA/cm^2^)	Polarization Resistance R_p_ (kΩ·cm^2^)	Corrosion Inhibition Efficiency η (%)
NaCl	−730 ± 4	−734 ± 7	1.3 ± 0.6	22 ± 9	-
Na_2_SiO_3_	−666 ± 11	−658 ± 21	0.4 ± 0.1	73 ± 26	69.2
Na_2_SiO_3_/MnSO_4_·H_2_O	−780 ± 17	−773 ± 2	0.18 ± 0.02	126 ± 7	86.1
Na_2_SiO_3_/NH_4_VO_3_	−854 ± 7	−862 ± 19	0.005 ± 0.002	545 ± 133	99.6
Na_2_SiO_3_/MnSO_4_·H_2_O/NH_4_VO_3_	−833 ± 5	−808 ± 8	0.004 ± 0.002	660 ± 62	99.7

## Data Availability

The original contributions presented in the study are included in the article, further inquiries can be directed to the corresponding author.
